# Supplementary material for: Retrospective analysis of cervical screening abnormalities in women with type 3 transformation zone without visible lesions
Source: PeerJ. 2025 Nov 27;13:e20396. doi: 10.7717/peerj.20396 (PMC12665263; doi:10.7717/peerj.20396)
Supplement: Supplemental Information 7 [file peerj-13-20396-s007.docx]

# Firth Penalized Logistic Regression Results with Cytology and HPV Interaction Terms for Predicting HSIL+

| Term | Estimate | Std_Error | CI_Lower | CI_Upper | ChiSq | OR | OR_CI_Lower | OR_CI_Upper |
| --- | --- | --- | --- | --- | --- | --- | --- | --- |
| (Intercept) | -5.1874 | 1.4182 | -10.0237 | -3.2616 | Inf | 0.0056 | 0.0000 | 0.0383 |
| Cytology(ASCUS) | 1.4169 | 1.5558 | -1.1085 | 6.3487 | 1.0835 | 4.1243 | 0.3301 | 571.7490 |
| Cytology(LSIL) | 1.4262 | 1.6411 | -1.5210 | 6.4159 | 0.8927 | 4.1629 | 0.2185 | 611.4909 |
| Cytology (ASC-H/HSIL/AGC) | 2.5248 | 1.6506 | -0.4408 | 7.5222 | 2.8211 | 12.4884 | 0.6435 | 1,848.6298 |
| HPV (Non-HPV 16/18) | 1.1857 | 1.4340 | -0.8042 | 6.0331 | 1.0197 | 3.2730 | 0.4474 | 417.0057 |
| HPV (HPV 16/18) | 1.7154 | 1.4342 | -0.2753 | 6.5630 | 2.5810 | 5.5589 | 0.7593 | 708.3937 |
| Cytology(ASCUS):HPV(Non-HPV 16/18) | -1.4653 | 1.5846 | -6.4195 | 1.1325 | 1.1013 | 0.2310 | 0.0016 | 3.1034 |
| Cytology(LSIL):HPV(Non-HPV 16/18) | -1.4859 | 1.6869 | -6.5137 | 1.5514 | 0.9071 | 0.2263 | 0.0015 | 4.7181 |
| Cytology(ASC-H/HSIL/AGC):HPV(Non-HPV 16/18) | 0.7518 | 1.6758 | -4.2664 | 3.7685 | 0.1778 | 2.1208 | 0.0140 | 43.3150 |
| Cytology(ASCUS):HPV(HPV 16/18) | -1.5765 | 1.6199 | -6.5596 | 1.1018 | 1.2117 | 0.2067 | 0.0014 | 3.0096 |
| Cytology(LSIL):HPV(HPV 16/18) | -0.7874 | 1.7247 | -5.8488 | 2.3187 | 0.2287 | 0.4550 | 0.0029 | 10.1625 |
| Cytology(ASC-H/HSIL/AGC):HPV(HPV 16/18) | 0.4363 | 1.6933 | -4.5965 | 3.4880 | 0.0621 | 1.5470 | 0.0101 | 32.7204 |
