# Supplementary material for: Retrospective analysis of cervical screening abnormalities in women with type 3 transformation zone without visible lesions
Source: PeerJ. 2025 Nov 27;13:e20396. doi: 10.7717/peerj.20396 (PMC12665263; doi:10.7717/peerj.20396)
Supplement: Supplemental Information 8 [file peerj-13-20396-s008.docx]

# P-values for Main and Interaction Effects in Firth Logistic Regression Model Predicting HSIL+

| Term | P_Value |
| --- | --- |
| (Intercept) | 0.00000 |
| Cytology(ASCUS) | 0.29791 |
| Cytology(LSIL) | 0.34475 |
| Cytology(ASC-H/HSIL/AGC) | 0.09303 |
| HPV(Non-HPV 16/18) | 0.31259 |
| HPV(HPV 16/18) | 0.10815 |
| Cytology(ASCUS):HPV(Non-HPV 16/18) | 0.29398 |
| Cytology(LSIL):HPV(Non-HPV 16/18) | 0.34089 |
| Cytology(ASC-H/HSIL/AGC):HPV(Non-HPV 16/18) | 0.67324 |
| Cytology(ASCUS):HPV(HPV 16/18) | 0.27099 |
| Cytology(LSIL):HPV(HPV 16/18) | 0.63248 |
| Cytology(ASC-H/HSIL/AGC):HPV(HPV 16/18) | 0.80318 |
