# Supplementary material for: Retrospective analysis of cervical screening abnormalities in women with type 3 transformation zone without visible lesions
Source: PeerJ. 2025 Nov 27;13:e20396. doi: 10.7717/peerj.20396 (PMC12665263; doi:10.7717/peerj.20396)
Supplement: Supplemental Information 9 [file peerj-13-20396-s009.docx]

Cross-classification of HSIL+ detection by ECC and cervical biopsy in women with type 3 transformation zone and negative colposcopy (N = 4,648)

|  | biopsies | |
| --- | --- | --- |
|  | HSIL+  N = 87 | HSIL-/Not performed  N = 4,561 |
| ECC |  |  |
| HSIL+（N=91） | 34 | 57 |
| HSIL-（N=4,557） | 53 | 4,504 |
