# Supplementary material for: Retrospective analysis of cervical screening abnormalities in women with type 3 transformation zone without visible lesions
Source: PeerJ. 2025 Nov 27;13:e20396. doi: 10.7717/peerj.20396 (PMC12665263; doi:10.7717/peerj.20396)
Supplement: Supplemental Information 10 — CIN, cervical intraepithelial neoplasia. [file peerj-13-20396-s010.docx]

| No. | Age | Gravidity | Parity | Menopause | TCT | HPV | HPV persistence | History of cervical neoplasia | TestType | Histologic Diagnosis |
| --- | --- | --- | --- | --- | --- | --- | --- | --- | --- | --- |
| 1 | 48 | 6 | 1 | Yes | ASCUS | Other High-risk HPV(Non-HPV16/18) | 3 years | CINI treated with cervical conization | ECC+2Biopsies | ECC:SCC;  Biopsy:chronic cervicitis. |
| 2 | 49 | 3 | 1 | No | LSIL | Other High-risk HPV(Non-HPV16/18) | Unknown | No | ECC+3Biopsies | ECC: SCC;  Biopsy:CINI. |
| 3 | 35 | 3 | 2 | No | ASC-H | HPV16 | Unknown | No | ECC | ECC: adenocarcinoma |
| 4 | 49 | 4 | 1 | No | NILM | HPV53 | 3 year | No | ECC+2Biopsies | Biopsy: SCC;  ECC:CIN III. |
| 5 | 31 | 0 | 0 | No | ASCUS | HPV51 | 1 years | CINII treated with cervical conization | ECC+1Biopsy | Biopsy:SCC:  ECC:CIN III. |
