# Supplementary material for: Retrospective analysis of cervical screening abnormalities in women with type 3 transformation zone without visible lesions
Source: PeerJ. 2025 Nov 27;13:e20396. doi: 10.7717/peerj.20396 (PMC12665263; doi:10.7717/peerj.20396)
Supplement: Supplemental Information 11 [file peerj-13-20396-s011.docx]

# STROBE Checklist for Cross-Sectional Study

## Title and Abstract

1. 1a. The study design is clearly indicated as a 'cross-sectional study' in both the title and abstract.
2. 1b. The abstract provides a clear and balanced summary, including background, objectives, methods, key results, and conclusion.

## Introduction

1. 2. Background rationale is presented in the Introduction, highlighting the clinical dilemma of managing women with abnormal screening but negative colposcopy and TZ3.
2. 3. Objectives are clearly stated in the last paragraph of the Introduction, including identifying HSIL+ predictors and defining risk thresholds.

## Methods

1. 4. The study design is described early in the 'Materials and Methods' as a cross-sectional study.
2. 5. The setting is described: Hunan Provincial Maternal and Child Health Care Hospital, 2021–2024.
3. 6a. Eligibility criteria include sexually active, not pregnant, intact uterus, no prior cervical cancer or pelvic radiation.
4. 7. Variables are clearly defined: outcomes (HSIL+), exposures (HPV genotype, cytology), and confounders (age, parity, menopause).
5. 8. Data sources and measurement methods are explained for cytology, HPV, colposcopy, ECC, and biopsy.
6. 9. Bias is addressed in limitations, including retrospective design and TZ3 anatomical constraints.
7. 10. Study size is based on inclusion/exclusion criteria applied to the screened cohort, shown in Figure 1.
8. 11. Quantitative variables such as age were treated both continuously and categorically.
9. 12a. Statistical methods include univariate and multivariate logistic regression, RCS modeling.
10. 12b. Interaction analysis used Firth logistic regression with cytology and HPV genotype.
11. 12c. Missing data were addressed through complete-case analysis.
12. 12d. Not applicable (no sampling strategy).
13. 12e. No sensitivity analyses were conducted.

## Results

1. 13a. Numbers at each stage are detailed in Figure 1, including exclusions and final sample.
2. 13b. Exclusion reasons include lack of ECC, visible lesions, incomplete data.
3. 13c. Flow diagram (Figure 1) included.
4. 14a. Participant characteristics listed in Table 1.
5. 14b. Missing data explained; large exclusions due to missing HPV or cytology data.
6. 15. Outcome data (HSIL+) presented across subgroups (Tables 2–6).
7. 16a. Unadjusted and adjusted odds ratios reported with 95% confidence intervals.
8. 16b. Age stratified into <55 and ≥55 years and further 5-year bands.
9. 16c. Interpretation based on clinical thresholds (e.g., 4% CIN3+ per ASCCP).
10. 17. Additional analyses include interaction terms and biopsy yield comparisons.

## Discussion

1. 18. Key results highlighted in the opening of Discussion.
2. 19. Limitations discussed, including generalizability and difficulty detecting lesions in TZ3.
3. 20. Interpretation considers limitations and relevance to clinical guidelines.
4. 21. Generalizability limited to similar high-volume screening settings.

## Other Information

22. Funding: supported by internal grants. No AI tools used in study design, data analysis, or interpretation
